# Discrete element modelling and mechanical properties and cutting experiments of *Caragana korshinskii* Kom. stems

**DOI:** 10.3389/fpls.2024.1457243

**Published:** 2024-10-31

**Authors:** Cao Qingqiu, Zhang Shengwei, Li Tao, Zhai Gaixia, Yuan Hongfang

**Affiliations:** ^1^ College of Biological and Agricultural Engineering, Jilin University, Changchun, China; ^2^ Research Center, Hohhot Branch of Chinese Academy of Agricultural Mechanization Sciences, Hohhot, China

**Keywords:** discrete element method, stem of *Caragana korshinskii* Kom., mechanical properties, parameter calibration, stem cutting

## Abstract

The forage crop *Caragana korshinskii* Kom. is of high quality, and the biomechanical properties of its plant system are of great significance for the development of harvesting equipment and the comprehensive utilisation of crop resources. However, the extant research on the biomechanical properties of *Caragana korshinskii* Kom. is inadequate to enhance and refine the theoretical techniques for mechanised harvesting. In this study, we established a discrete element model of CKS based on the Hertz-Mindlin bonding contact model. By combining physical experiments and numerical simulations, we calibrated and validated the intrinsic and contact parameters. The Plackett-Burman design test was employed to identify the significant factors influencing bending force, and the optimal parameter combination for these factors was determined through response surface analysis. When the shear stiffness per unit area was 3.56×10^9^ Pa, the bonded disk scale was 0.93 mm, the normal stiffness per unit area was 9.68×10^9^ Pa, the normal strength was 5.62×10^7^ Pa, the shear strength was 4.27×10^7^ Pa, the discrete element numerical simulation results for three-point bending, radial compression, axial tension, and shear fracture exhibited a maximum failure force error of 3.32%, 4.37%, 4.87% and 3.74% in comparison to the physical experiments. In the cutting experiments, a smaller radial angle between the tool edge and the stem resulted in less damage to the cutting section, which was beneficial for the smoothness of the stubble after harvesting and the subsequent growth of the stem. The discrepancy in cutting force between the physical and numerical simulations was 3.89%, and the *F*-*x* (force versus displacement) trend was consistent. The multi-angle experimental validation demonstrated that the discrete element model of CKS is an accurate representation of the real biomechanical properties of CKS. The findings offer valuable insights into the mechanisms underlying crop-machine interactions.

## 1 Introduction

*Caragana korshinskii* Kom., a member of the Leguminosae family, is a shrub that is widely distributed in Inner Mongolia, Ningxia, Shanxi, Shaanxi, and Gansu provinces of China, as well as in Mongolia (Zhang et al., 2010; Yang et al., 2013). It can be utilised as forage and an ecological protection species, possessing considerable economic and ecological value (Liang et al., 2019; Roy et al., 2021). In order to promote population renewal and sustainable growth, stem cutting operations must be performed every three to five years, in accordance with the plant’s growth characteristics and agronomic requirements (Wang et al., 2013; Gao et al., 2021). The morphology of the stubble section after cutting is a pivotal factor influencing the rejuvenation of new branches (Gao et al., 2021). At present, the biomechanical properties of CKS are not sufficiently researched to support the development of harvesting equipment. This results in the residual stems exhibiting a lack of smoothness following harvesting, which in turn affects their subsequent growth and causes a significant waste of biological resources.

To provide evidence for the design of harvesting machinery, the rejuvenation of plants, and the increase in crop yield based on the mechanical properties of plant stems, many researchers have conducted extensive studies on the biomechanical properties of stems in crops such as barley, corn, wolfberry, tea, hemp, and other crops. These studies have played a significant role in guiding agricultural production activities (Khan et al., 2010; Liu et al., 2012; Aydin and Arslan, 2018; Chen et al., 2019; Ma et al., 2020; Guo et al., 2021; Luo et al., 2022; Xia et al., 2022). However, conventional physical testing techniques for stem biomechanical properties are only capable of examining the macroscopic aspects and are unable to elucidate the failure mechanisms within the internal biological tissues of the stems at the microscopic level. This limitation impedes a comprehensive investigation into the dynamic interaction between the stems and the cutting devices.

The discrete element method has been employed extensively in the construction of plant stem models in light of the accelerated advancement of computers (Schramm and Tekeste, 2022). (Fu et al., 2023) employed a combination of physical measurements and computer simulation to ascertain the parameters of micro-crushing of corn stalk pulverisation, thereby providing technical support for the development of stalk micro-crushing equipment. (Leblicq et al., 2016a) employed the discrete element method (DEM) to numerically simulate the compression and bending processes (Leblicq et al., 2016b) of corn stem, thereby demonstrating the efficacy of DEM in characterising the mechanical properties of the stem. (Huan et al., 2022) investigated the cutting and flinging process of king grass stalks in mechanical harvesting with a discrete element method, elucidating the mechanism of interaction between king grass stalks and harvesting machinery. (Lei et al., 2022) calibrated the discrete element numerical simulation parameters of the tail stem and tail leaves of crushed sugarcane (STL) by combining physical experiments and numerical simulation with an optimised design, and verified the reliability of the calibrated parameters. (Hu et al., 2023) calibrated the parameters of the DEM model of ramie, verified the accuracy of the ramie DEM model, and provided theoretical support for the simulation of ramie stalk peeling. (Jiang et al., 2021) established a model of a cotton stalk using the discrete element method and identified the optimal parameter combinations, which facilitated the simulation of the shredding apparatus and the analysis of the material preparation. (Wang et al., 2020) established the DEM of citrus fruit stalks, which is capable of accurately simulating the bending and shearing actions of citrus fruit stalks. The results of this study are of considerable importance for the optimisation of the end-effector of harvesting robots for citrus fruit stalks. (Liu et al., 2022) established a DEM model of taro using a combination of physical tests and numerical modelling. The model was verified through clamping and pulling experiments, thereby providing a reference for optimising the taro harvester. The aforementioned studies demonstrate the efficacy of the DEM as a methodology for simulating crop stalks. By integrating physical experimentation with computational modelling, it is feasible to delve deeper into the internal micro-mechanical attributes and failure mechanisms of plant stems. In comparison to conventional experimental testing techniques, computer-based numerical simulation offers a number of advantages, including the ability to generate accurate and reliable test results, facilitate repetition, and verify the accuracy of traditional experiments. However, there is a paucity of literature on the DEM model of CKS, and the field of numerical simulation research in this area remains largely uncharted territory.

The objective of this study was to conduct a series of mechanical tests on CKS, including three-point bending, radial compression, axial tension, and shear fracture tests. A discrete element model of CKS was established based on DEM, calibrated and validated. The optimal parameter combination for the CKS model was determined, and the maximum failure force and *F*-*x* curve trends were compared between physical experiments and numerical simulations. Cutting experiments were designed to analyse the cutting force of physical and numerical simulation tests to verify the correctness of the discrete element model and to provide a correct and reliable CKS discrete element model for the DEM numerical simulation of the operation process of the *Caragana korshinskii* Kom. harvesting equipment.

## 2 Materials and methods

### 2.1 Experimental materials

#### 2.1.1 Experimental samples

**Figure 1:**
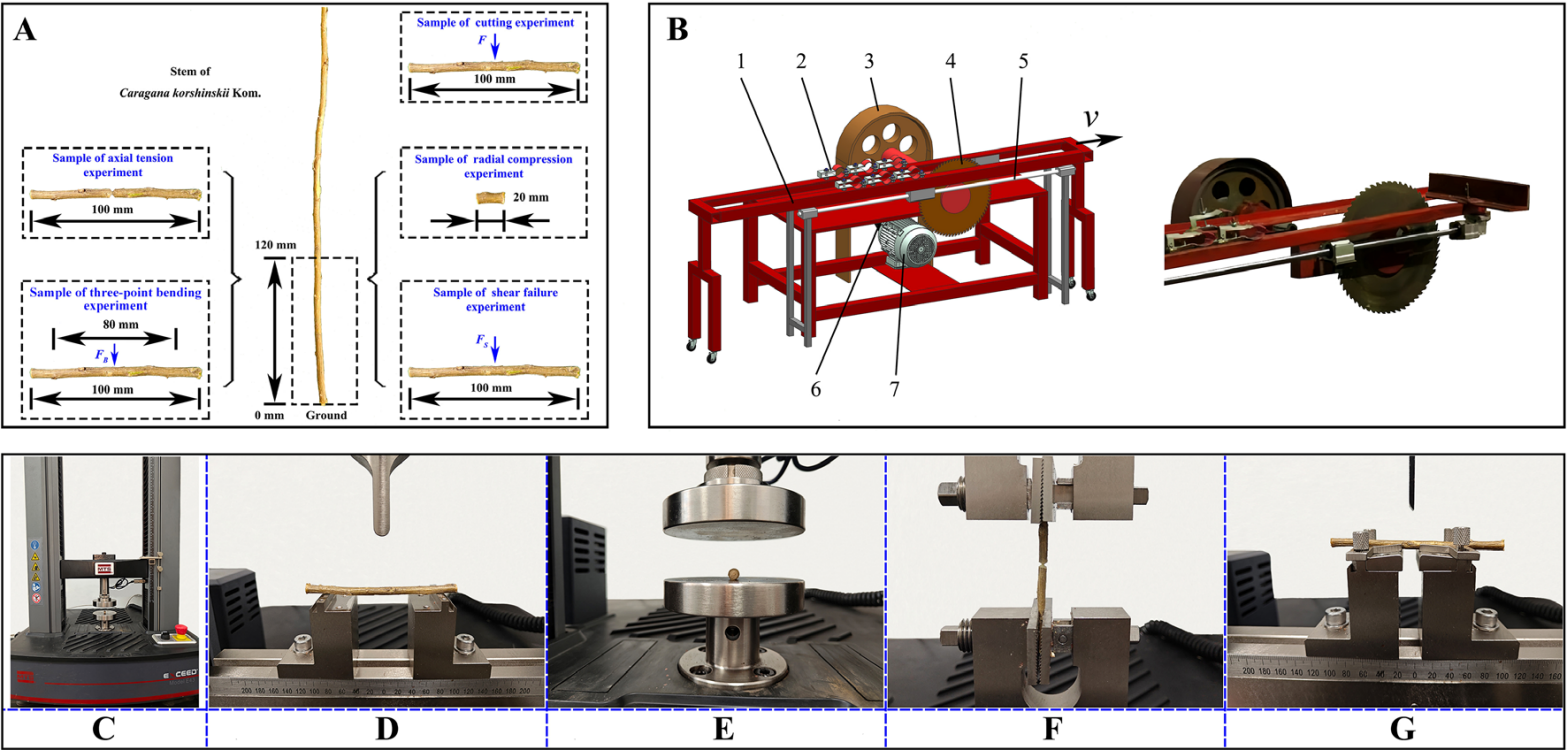
Experimental sample and device. **(A)** Experiment sample preparation; **(B)** Cutting experiment device. 1. Movable frame 2. Fixture 3. Pulley 4. Circular cutter 5. Guide rails 6. Belt 7. Motor; **(C)** MTS Exceed E43; **(D)** Three-point bending experiment; **(E)** Radial compression experiment; **(F)** Axial tension experiment; **(G)** Shear fracture experiment.

*Caragana korshinskii* Kom. was used as the experimental material. Samples were gathered utilising the five-point sampling method (Ren et al., 2021) from Fanjiayao Village, Helinger County, Hohhot, Inner Mongolia Autonomous Region, at the experimental field of the Hohhot branch of the Chinese Academy of Agricultural Mechanization Sciences (40°16′N, 111°44′E). Only specimens of *Caragana korshinskii* Kom. that exhibited no obvious signs of pests or diseases were selected. The stems were pruned to remove any superfluous branches and leaves, leaving only the requisite stems for the production of experimental samples. In consideration of regional characteristics, the cutting operation of CKS was primarily concentrated between 0 mm and 120 mm above the ground (Chen et al., 2020), which was intercepted and the stems were cut into 100 mm and 20 mm. The 100 mm segments were employed in three-point bending, axial tension, shear fracture, and cutting tests, whereas the 20 mm segments were utilised in compression tests, as illustrated in **Figure 1A**. To ensure the accuracy of the test data, all CKS samples were maintained at a moisture content of between 35.6% and 44.3% (wet basis). The diameter of the CKS utilised in the physical experiments was 6 ± 0.47 mm and the diameter of the discrete element model of the CKS established in this study was set to 6 mm. To guarantee the precision of the experiments, all physical tests were conducted within 48 hours.

The CKS were subjected to four distinct tests: three-point bending (**Figure 1D**), radial compression (**Figure 1E**), axial tension (**Figure 1F**), and shear fracture (**Figure 1G**). These tests were conducted using an MTS Exceed E43 universal testing machine, as illustrated in **Figure 1C**. During the course of the tests, the force, displacement, and force-displacement (*F*-*x*) curves of the samples were recorded using the computer that was connected to the testing machine. The testing machine is equipped with a force sensor with a maximum capacity of 10 kN, and the testing speed ranges from 0.001 to 500 mm/min. The four distinct test procedures were conducted by utilising different fixtures. The loading speeds for the three-point bending, radial compression, and shear fracture tests were set at 15 mm/min, while the loading speed for the axial tension test was set at 2 mm/min. Each test method was performed on ten samples.

#### 2.1.2 DEM model

##### 2.1.2.1 Contact model

**Figure 2:**
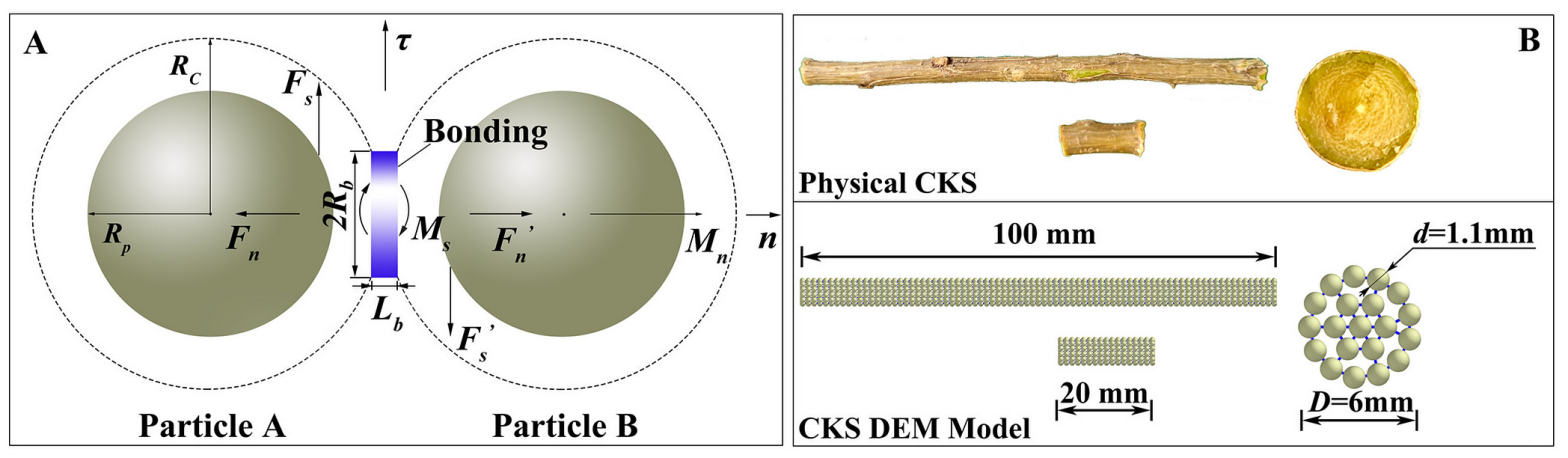
The discrete element model of CKS. **(A)** Hertz-Mindlin contact model with the bonding contact; **(B)** The size of physical CKS and the DEM Model of CKS.


According to previous studies, the biomechanical properties of CKS could be better reflected by choosing the Hertz-Mindlin (no-slip) and standard rolling friction models to study the interaction between CKS particles and steel (Liu et al., 2018), on the basis of these models, the Hertz-Mindlin bonding contact model was used to describe the interactions between CKS particles, with parallel bonds representing the interaction forces between two particles, as illustrated in **Figure 2A**. *F_n_* and *F_n_’* are the normal forces of the bond, *F_s_* and *F_s_’* are the tangential forces of the bond; *M_n_* and *M_s_* are the normal and tangential moments of the bond, respectively. where *k_n_* and *k_s_* are the normal and shear stiffness per unit area, *V_n_*, *V_t_*, ***ω*** *_n_*, ***ω*** *_t_* are the normal and tangential velocities, and the normal and tangential angular velocities, respectively; *L_b_* is the length of the bond; *R_b_* is the bonded disk scale; *R_p_* is the radius of the particles; and *R_c_* is the radius of the contact between the particles. *R_c_* is 1.2 to 2 times of *R_p_* (Zhang et al., 2019), this spacing can help to generate the parallel bond better. The force acting on the particle and the action force were determined using (Equation 1) (Jiménez-Herrera et al., 2018).

(1) $$\Delta F_{n}=\;\delta F_{n}^{'}=-k_{n}A_{b}V_{n}\Delta t$$

$$\Delta F_{s}=\;\delta F_{s}^{'}=-k_{s}A_{b}V_{t}\Delta t$$

$$\Delta M_{n}=\;-k_{s}I\omega_{n}\Delta t$$

$$\Delta M_{s}=\;-k_{n}I_{P}\omega_{t}\Delta t$$

Where,


(2) $$A_{b}=\;\pi R_{b}^{2}$$

$$I=\;\frac{1}{2}\pi R_{b}^{4}$$

$$I_{P}=\;\frac{1}{4}\pi R_{b}^{4}$$

Where *I*, *I_P_*, and *A_b_* are the cross-sectional moment of inertia, polar moment of inertia, and cross-sectional area of the bond, respectively. The forces *F* and moments *M* (Equation 2) acting in the normal and shear directions vary due to the relative normal and shear displacements of the sphere as well as the normal and shear rotations of the sphere. Both *F_s_* and *F_s_’* act on the bond and the bond breaks down when these forces exceed a critical value, as shown in (Equation 3)


(3) $$\sigma_{\text{max}}<\frac{-F_{n}}{A_{b}}+\frac{2M_{n}}{J}R_{b}$$

$$\tau_{\text{max}}<\frac{-F_{s}}{A_{b}}+\frac{2M_{s}}{J}R_{b}$$

Where *σ*_max_ and *τ*_max_ are the normal and tangential critical stresses, respectively.

##### 2.1.2.2 The DEM model of experimental materials

In DEM numerical simulations, the multi-sphere method had been a commonly employed technique for establishing irregular particle models (Liu et al., 2018; Zeng and Chen, 2019; Liao et al., 2020; Fan et al., 2022). In the context of discrete element numerical simulations of non-spherical particles, the multi-sphere packing model offered a number of advantages, including a relatively fast computational speed and a straightforward approach to contact judgment. However, as the number of filling particle elements increased, the simulation time increased significantly, necessitating the use of a reasonable number of filling particle elements (Kruggel-Emden et al., 2008; Cabiscol et al., 2018). The modeling process of the CKS was as follows: (1) Established the geometric model of the CKS used in the experiments in Unigraphics NX; (2) Imported the model into ICEM CFD software for structured meshing to obtain the coordinates of the centroids of each mesh element; (3) Used the element particle model in EDEM to generate the DEM model of the experimental CKS by matching the mesh centroid coordinates to the particle center coordinates. The diameter and length of the DEM model were maintained in accordance with the specifications of the physical experiments. A total of 1,660 standard spherical particles with a length of 100 mm and 340 standard spherical particles with a length of 20 mm were generated, as illustrated in **Figure 2B** (The methodology employed to define the particle parameters of the DEM model was outlined in the **Supplementary Material**.).

### 2.2 Determination of intrinsic parameters of the DEM model

The intrinsic parameters of the DEM model include density, Poisson’s ratio, and shear modulus.. The density *ρ* was determined by standard methods, with a value of 1014 kg·m^-3^ (ASTM D854-10, 2010), and the Poisson’s ratio was set to 0.4 (Madison et al., 2010). The shear modulus *G* was calculated through a three-point bending test, as illustrated in **Figure 1D**. In the three-point bending test, the CKS was positioned on the test bench with a sample length of 100 mm and a diameter of 5.93 ± 0.26 mm. The distance between the two support points was 80 mm, and a cylindrical indenter with a diameter of 10 mm was applied at a rate of 15 mm/min. The bending force and displacement (*F_B_-x*) curve was recorded by the MTS Exceed E43. The elastic modulus *E* is calculated using (Equation 4), and the shear modulus *G* is then obtained using (Equation 5), where *y* is the deflection (displacement), *F_B_* is the bending force, and *S* is the spanning distance between the support points. (scale length), *E* is the modulus of elasticity, *J* is the moment of inertia of the CKS cross-section, *dF/dy* is the slope of the *F_B_-x* curve, and *μ* is the Poisson’s ratio of the CKS.


(4) $$y=\;\frac{F_{B}S^{3}}{48EJ}$$

$$J=\;\frac{\pi }{64}D^{4}$$

(5) $$G=\;\frac{E}{2\left(1+\mu \right)}$$

The three-point bending experiment was conducted ten times. The average maximum bending force *F_B_* was found to be 115.67 N, while the average modulus of elasticity *E* was calculated to be 6.46 × 10^9^ Pa. The shear modulus *G* was determined to be 2.31 ×10^9^ Pa.

### 2.3 Determination of other parameters of the DEM model

The study established the contact parameters of the material and the parallel bond parameters of the DEM model. The methods for determining the material’s contact parameters were relatively well established; detailed measurement methods could be found in the **Supplementary Material** and would not be discussed here. In the Hertz-Mindlin bonding contact model of the DEM, five parallel bond parameters were used to characterise the interaction forces between two CKS discrete element particles: normal stiffness per unit area, shear stiffness per unit area, normal strength, shear strength, and bonded disk scale. The ranges for these five parameters were based on the findings of previous research (Luo et al., 2019; Liao et al., 2020). In order to ascertain the requisite level values for the parallel bond parameters, preliminary experimental studies were conducted. **Table 1** presents the values for the contact and parallel bond parameters.

**Table 1:** Parameters required for DEM simulation.

Parameter	Symbol	Parameter levels		
		Low (-1)	Medium (0)	High (+1)
Coefficient of restitution (CKS-Steel)	*x*_1_	0.22	0.46	0.70
Coefficient of static friction (CKS-Steel)	*x*_2_	0.12	0.31	0.50
Coefficient of rolling friction (CKS-Steel)	*x*_3_	0.15	0.25	0.35
Coefficient of restitution (CKS-CKS)	*x*_4_	0.20	0.4	0.60
Coefficient of static friction (CKS-CKS)	*x*_5_	0.10	0.23	0.36
Coefficient of rolling friction (CKS-CKS)	*x*_6_	0.20	0.34	0.48
Normal stiffness per unit area/(N·m^-3^)	*k_n_*	1.00×10^9^	5.50×10^9^	1.00×10^10^
Shear stiffness per unit area/(N·m^-3^)	*k_s_*	1.00×10^9^	5.50×10^9^	1.00×10^10^
Normal strength/(Pa)	*σ*_max_	1.00×10^6^	5.05×10^7^	1.00×10^8^
Shear strength/(Pa)	*τ*_max_	1.00×10^6^	5.05×10^7^	1.00×10^8^
Bonded disk scale/(mm)	*R_b_*	0.9	1.0	1.1

### 2.4 Optimized design of discrete element model parameters

Firstly, numerical simulation tests were conducted in the EDEM software, utilising the three-point bending test force *F_B_* as an indicator. The Plackett-Burman design test (Zhang et al., 2023) was employed to identify the significant factors affecting the outcome. Secondly, a mathematical model of *F_B_* and the significant factors was established using a response surface analysis methodology, and the optimal model parameter combination was established by solving the mathematical model. Finally, numerical simulations of three-point bending, radial compression, axial tension, and shear fracture were conducted using the DEM model with the optimal parameters. The maximum simulated failure forces and the trends of the force-displacement curves were compared with the physical test results to verify the accuracy of the DEM model of CKS.

#### 2.4.1 Plackett-Burman design experiment

In this study, the Plackett-Burman module in Design-Expert software was employed to evaluate the effect of eleven factors on the bending force *F_B_* as the response value. Each factor was assigned both a high and a low level. A total of 15 experimental runs were conducted, with three centre points established at the outset. The experimental design and results are presented in **Table 2**. Additionally, a Pareto chart was established.

**Table 2:** Design and results of Plackett-Burman design test.

No.	*x*_1_	*x*_2_	*x*_3_	*x*_4_	*x*_5_	*x*_6_	*k_n_*	*k_s_*	*σ*_max_	*τ*_max_	*R_b_*	*F_B_*
1	1	1	-1	1	1	1	-1	-1	-1	1	-1	48.1
2	-1	1	1	-1	1	1	1	-1	-1	-1	1	105.1
3	1	-1	1	1	-1	1	1	1	-1	-1	-1	138.9
4	-1	1	-1	1	1	-1	1	1	1	-1	-1	148.3
5	-1	-1	1	-1	1	1	-1	1	1	1	-1	141.4
6	-1	-1	-1	1	-1	1	1	-1	1	1	1	141.3
7	1	-1	-1	-1	1	-1	1	1	-1	1	1	138.2
8	1	1	-1	-1	-1	1	-1	1	1	-1	1	144.7
9	1	1	1	-1	-1	-1	1	-1	1	1	-1	103.9
10	-1	1	1	1	-1	-1	-1	1	-1	1	1	182.7
11	1	-1	1	1	1	-1	-1	-1	1	-1	1	81.8
12	-1	-1	-1	-1	-1	-1	-1	-1	-1	-1	-1	43.7
13	0	0	0	0	0	0	0	0	0	0	0	180.1
14	0	0	0	0	0	0	0	0	0	0	0	198.3
15	0	0	0	0	0	0	0	0	0	0	0	183.8

#### 2.4.2 Response surface experiment

Based on the results of the Plackett-Burman design experiment, the Box-Behnken module in Design-Expert software was employed to undertake response surface optimisation experiments. The optimal parameter combination for the three most significant factors was identified. A total of 15 experimental runs were conducted, with three centre points established. The experimental design and results are presented in **Table 3**. Subsequently, the central composite design experiment was employed to ascertain the optimal parameter combination for the remaining two factors. Three centre points were established during the course of the experiments, resulting in a total of nine experimental runs. The experimental design and results are presented in **Table 4**.

**Table 3:** Design and results of Box-Behnken design test.

No.	*k_n_*	*k_s_*	*R_b_*	*F_B_*
1	-1	-1	0	154.3
2	1	-1	0	142.5
3	-1	1	0	196.1
4	1	1	0	228.2
5	-1	0	-1	123.1
6	1	0	-1	121.0
7	-1	0	1	163.9
8	1	0	1	202.3
9	0	-1	-1	60.1
10	0	1	-1	178.9
11	0	-1	1	167.2
12	0	1	1	213.4
13	0	0	0	171.7
14	0	0	0	177.5
15	0	0	0	180.8

**Table 4:** Design and results of central composite design test.

No.	*σ*_max_	*τ*_max_	*F_B_*
1	-1	-1	94.4
2	1	-1	113.7
3	-1	1	112.3
4	1	1	157.8
5	1.41421	0	138.2
6	0	1.41421	145.4
7	0	0	120.9
8	0	0	118.6
9	0	0	115.7

### 2.5 Cutting experiments

A cutting test bench was employed for both physical and numerical simulation experiments, as illustrated in **Figure 1B**. The power generated by the motor was conveyed through a belt to operate the circular cutter. The CKS was affixed to a movable frame via a fixture, with a traction motor positioned on the traction side of the movable frame. This configuration enabled the CKS to be transported horizontally along the guide rails to the circular cutter. A high-precision sensor connected the traction motor and the movable clamping device transmited the force data to a computer for analysis. During the test, the circular cutter had a diameter of 400 mm, a rotational speed of 600 rpm, and a material feed rate of 0.6 m/s. The numerical simulation experiments were conducted under identical operating conditions to those of the physical experiments.

## 3 Results and discussion

### 3.1 Plackett-Burman design experiment analysis

**Figure 3:**
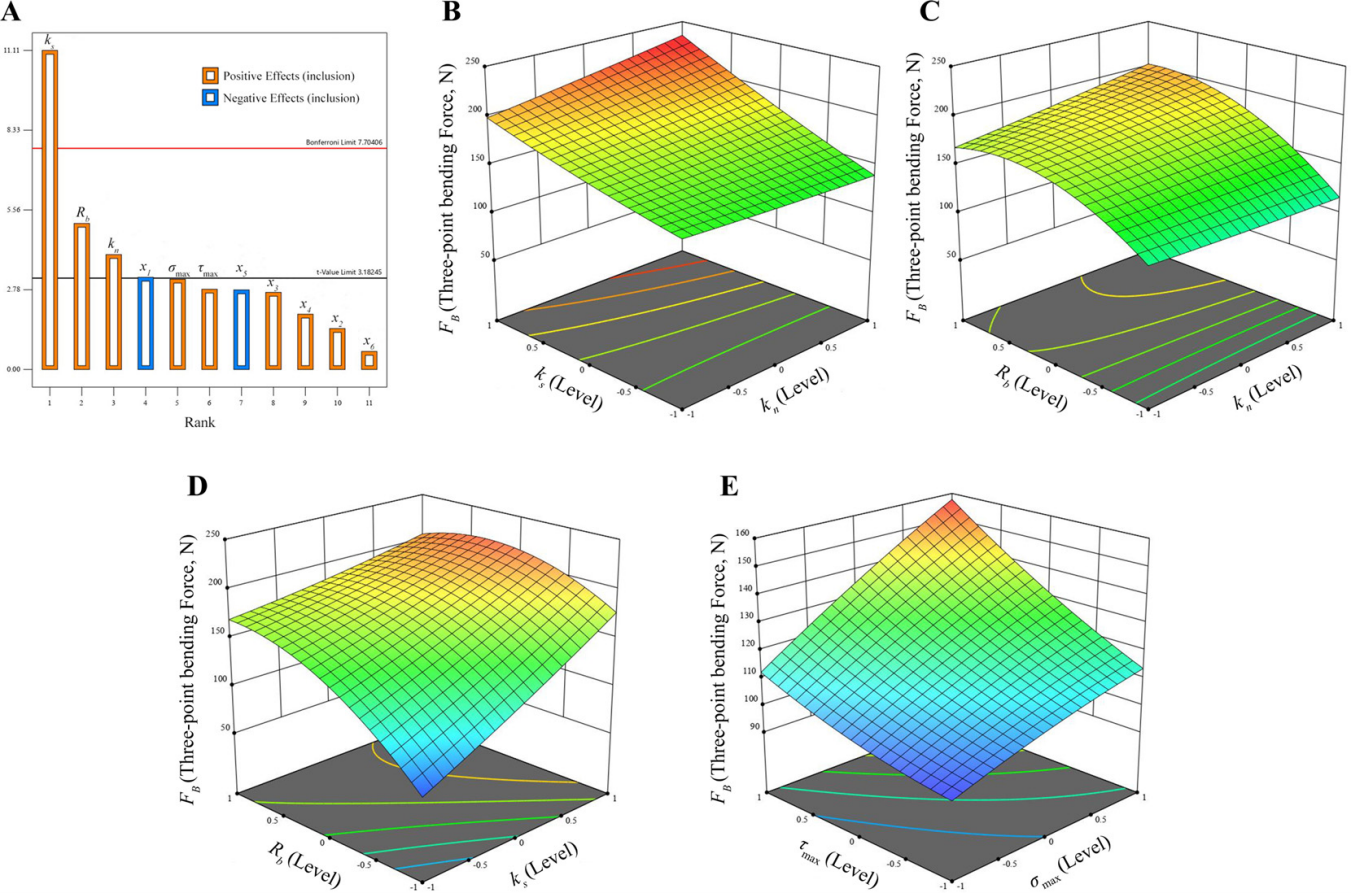
Pareto chart and the response surface analysis. **(A)** Pareto chart of significant parameters; **(B)** Effects of *k_s_* and *k_n_* on *F_B_*; **(C)** Effects of *R_b_* and *k_n_* on *F_B_*; **(D)** Effects of *R_b_* and *k_s_* on *F_B_*; **(E)** Effects of *σ*_max_ and *τ*_max_.

As illustrated in **Figure 3A**, when the contribution of the DEM parameters exceeds the t-value limit, a notable impact is observed on the response. Conversely, when the contribution of the parameters is below the t-value limit, the effect on the response is almost negligible (Xia et al., 2019). A positive effect indicates that as the level of the factor in question increases, the value of *F_B_* also increases. Conversely, a negative effect represents the opposite phenomenon. The effects on *F_B_*, in descending order, are as follows: *k_s_*, *R_b_*, *k_n_*, *σ*_max_, *τ*_max_, *x*_3_, *x*_2_, *x*_1_. The parameters that have a significant effect on *F_B_* are *k_s_*, *R_b_*, and *k_n_*. Furthermore, the effects of the three significant factors are positively correlated.

### 3.2 Response surface analysis

The Design-Expert software was employed to align the test outcomes of the Box-Behnken design with the fitting operation, thereby establishing a mathematical model of the bending force *F_B_* and the influencing factors, as illustrated in (Equation 6). Subsequently, the analysis of variance (ANOVA) was conducted, and the test protocols (factors not enumerated were assumed to be at the level of 0) and the outcomes are presented in **Table 3**.


(6) $$F_{B}=176.67+7.08k_{n}+36.56k_{s}+32.96R_{b}+10.98k_{n}k_{s}+10.13k_{n}R_{b}-18.15k_{s}R_{b}+0.64k_{n}^{2}+2.97k_{s}^{2}-24.73R_{b}^{2}$$

**Table 5** reveals that the model *P*-value is 0.0013, which is less than 0.01, indicating that the model is statistically significant. The *P*-value of the misfit term is 0.4805, which is greater than 0.05, suggesting that the misfit term is not significant. The coefficient of determination *R^2^* is 0.9775, and the coefficient of variation is 6.31%. The adjusted coefficient of determination *R2 adj* is 0.9370, indicating that the model has a high level of predictive power. The difference between the adjusted coefficient of determination and the prediction coefficient *R2 pre* is less than 0.2, which suggests that the model does not have a large block effect and is therefore highly predictive. It can be observed that both *k_s_* and *R_b_* have a highly significant effect on *F_B_* (*P* < 0.01), while *k_n_* has a significant effect on *F_B_* (*P* < 0.05). The remaining terms are not significant.

**Table 5:** ANOVA of the Box-Behnken design test.

Source of variance	Sum of Squares	Degree of freedom	Mean square	*F*-value	*P*-value
Model	24361.54	9	2706.84	45.45	0.0003**
*k_n_*	400.44	1	400.44	6.72	0.0487*
*k_s_*	10694.53	1	10694.53	179.58	< 0.0001**
*R_b_*	8692.21	1	8692.21	145.96	< 0.0001**
*k_n_k_s_*	481.8	1	481.8	8.09	0.0361*
*k_n_R_b_*	410.06	1	410.06	6.89	0.0469*
*k_s_R_b_*	1317.69	1	1317.69	22.13	0.0053**
*k_n_* ^2^	1.52	1	1.52	0.0255	0.8793
*k_s_* ^2^	32.5	1	32.5	0.5457	0.4933
*R_b_* ^2^	2258.72	1	2258.72	37.93	0.0016**
Residual	297.76	5	59.55		
Total	24659.3	14			

The response surface of the three significant factors affecting the *F_B_* (**Figure 3B**) demonstrates that the *F_B_* required for the numerical simulation tests of three-point bending increases in proportion to the values of *k_n_* and *k_s_*, in accordance with the theoretical basis presented in (Equation 1). In conjunction with **Table 5**, it is evident that *k_s_* exerts a more pronounced influence on *F_B_* than *k_n_*. This is due to the fact that when the model is subjected to bending forces, a pair of reversed tangential forces are generated internally, and an increase in *k_s_* enhances the bond’s capacity to resist deformation in the tangential direction (Wang et al., 2020). This, in turn, leads to enhanced tangential elastic recovery and stability, which improves inter-particle contact stresses and increases *F_B_* in order to break the bond. **Figure 3C** and **Figure 3D** demonstrate that an increased *R_b_* also results in a larger required *F_B_*, which is consistent with the theoretical basis of (Equations 1, 2). As indicated by the *P*-values of the three significant factors in **Table 5**, the order of influence on *F_B_* is *k_s_* > *R_b_* > *k_n_*, which aligns with the order of influence of the three factors as depicted in the Pareto chart (**Figure 3A**). The parallel bond parameter combinations were obtained by solving the response surface analysis of Design-Expert software. The results yielded the following values: *k_s_* is 3.56×10^9^ Pa, *R_b_* is 0.93 mm, *k_n_* is 9.68×10^9^ Pa.

It should be noted that the remaining two parameters may fall outside the preset range. Therefore, in order to determine the values of *σ*_max_ and *τ*_max_, the central composite design method was employed, utilising the optimal parameter combinations of *k_s_*, *R_b_*, and *k_n_* determined in the previous section. The mathematical model of the bending force *F_B_* with *σ*_max_ and *τ*_max_ is presented in Equation 7.


(7) $$F_{B}=118.4+16.17\sigma_{\text{max}}+15.47\tau_{\text{max}}+6.55\sigma_{\text{max}}\tau_{\text{max}}-1.49\sigma_{\text{max}}^{2}+2.60\tau_{\text{max}}^{2}$$

As the values of *σ*_max_ and *τ*_max_ must be treated as positive numbers, the non-compliant test items were excluded from further consideration. Subsequently, ANOVA was conducted, and the resulting test outcomes were presented in **Table 6**. The model *P*-value was 0.0229, which was less than 0.01, indicating that the model was significant. The *P*-value of the misfit term was 0.9964, which was greater than 0.05, demonstrating that the misfit term was not significant. The coefficient of determination *R* ^2^ was 0.9635, the coefficient of variation was 4.77%, and the adjustment coefficient *R* ^2^ *_adj_* was 0.9028. The difference between the prediction coefficient *R* ^2^ *_pre_* of 0.9179 and the value of 0.9028 was less than 0.2, indicating that the model did not have a large block effect and that it performed better in prediction. The values of *σ*_max_ and *τ*_max_ had a significant effect on *F_B_* (P < 0.05), while the remaining terms where not significant. The order of influence on *F_B_* was *σ*_max_ > *τ*_max_, which was consistent with the order of influence of the two factors corresponding to the Pareto chart (**Figure 3A**).

**Table 6:** ANOVA of the central composite design test.

Source of variance	Sum of Squares	Degree of freedom	Mean square	*F*-value	*P*-value
Model	3015.12	5	603.02	132.95	0.001**
*σ*_max_	1195.4	1	1195.4	263.55	0.0005**
*τ*_max_	1094.15	1	1094.15	241.23	0.0006**
*σ*_max_ *τ*_max_	171.61	1	171.61	37.83	0.0086**
*σ* ^2^_max_	7.02	1	7.02	1.55	0.3019
*τ* ^2^_max_	21.31	1	21.31	4.7	0.1187
Residual	13.61	3	4.54		
Total	3028.73	8			

Increasing *σ*_max_, *τ*_max_ can raise the thresholds of normal strength and shear strength of the bond, without altering the remaining parameters in (Equation 3). The sole means of achieving this is to augment the *F_B_*, and the bond will be rendered unstable when the *F_B_* surpasses the normal strength and shear strength of the bond. This is in accordance with the observed trend of increasing *σ*_max_, *τ*_max_, which subsequently results in an increase of *F_B_*, as illustrated in **Figure 3E**. The five parallel bond parameters of CKS were determined by response surface analysis of Design-Expert software and are as follows: *k_s_* is 3.56×10^9^ Pa, *R_b_* is 0.93 mm, *k_n_* is 9.68×10^9^ Pa, *σ*_max_ is 5.62×10^7^ Pa, and *τ*_max_ is 4.27×10^7^ Pa.

### 3.3 Validation of the correctness of the DEM model

#### 3.3.1 Analytical verification of shear failure experiments

**Figure 4:**
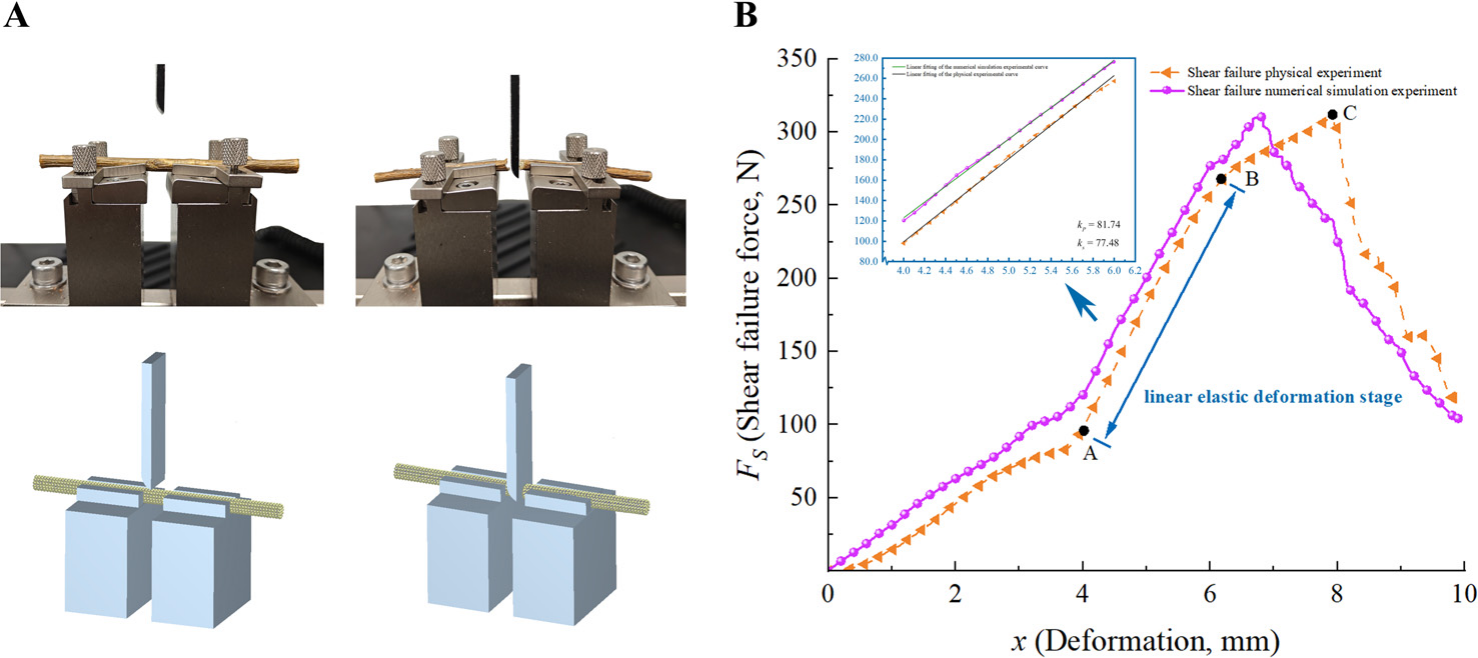
Analysis of shear fracture experiments. **(A)** The shear failure physical and numerical simulation experiments of CKS; **(B)** The *F_S_*-*x* curves of physical and numerical simulation experiments.

**Figure 4A** shows the shear failure physical and numerical simulation experiments of CKS. As illustrated in **Figure 4B**, the shear fracture process can be delineated into four distinct stages: elastic deformation, linear elastic deformation, shear failure, and fracture. The elastic deformation stage (*OA*) demonstrates a negligible variation in shear force with increasing displacement. This phenomenon can be attributed to the resilience of the phloem in the stem, where the load applied by the tool primarily compresses the stem without the blade cutting into it, resulting in elastic deformation of the stem (Guo et al., 2021). As the shear force increases, the process progresses to the linear elastic deformation stage (*AB*). In this phase, the shear force increases exponentially with displacement as the blade penetrates the stem, resulting in predominantly plastic deformation. Subsequently, at point *B*, the process transitions to the shear failure stage, wherein the rate of increase in shear force decelerates. This is due to the fact that, upon entering the plastic deformation stage, the original biological tissue structure of the stem is destroyed. The application of the tool results in the accumulation of fractured tissues, which in turn causes the shear force to continue increasing. However, the accumulated tissues are unable to attain the shear strength of the original biological structure, resulting in a lower rate of force increase in comparison to the plastic deformation stage. At point *C*, the maximum shear force is reached, after which there is a rapid decrease in shear force as the stem fractures, thereby marking the conclusion of the test.

A comparison of the *F_S_-x* curves derived from the numerical simulation and physical tests reveals that the rate of change in shear force as it transitions from the elastic deformation stage to the linear elastic deformation stage in the numerical shear fracture test is lower than in the physical shear fracture test. This is due to the fact that the DEM model of CKS simplifies the material by not distinguishing between the phloem and xylem, treating it as a homogeneous material. This results in a more gradual change in shear force in the numerical simulation. The discrepancy between the maximum shear force *F_S_* values obtained from the physical and numerical simulation tests is 3.74%, as presented in **Table 7**.

**Table 7:** The results of shear failure, axial tension and compression experiments.

No.	Shear failure experiments		Axial tension experiments				Compression experiments	
	Diameter (mm)	*F_S_* (N)	Diameter (mm)	Lengh (mm)	Displacement (mm)	*F_T_* (N)	Diameter (mm)	*F_C_* (N)
1	6.01	309.69	1.12	100.31	0.44	77.77	6.11	1208.92
2	5.53	257.84	1.11	102.27	0.41	67.65	6.08	1230.52
3	6.12	422.79	1.12	97.28	0.53	75.47	6.26	1446.25
4	5.80	278.16	1.10	100.95	0.51	96.08	5.97	1245.25
5	5.72	287.10	1.13	98.57	0.48	73.83	5.90	1130.48
6	6.36	397.36	1.08	99.21	0.55	72.34	6.31	1386.34
7	6.10	335.17	1.09	103.43	0.39	71.82	6.01	1307.92
8	5.98	332.48	1.06	101.49	0.42	70.59	5.83	1257.68
9	6.04	307.85	1.09	97.47	0.52	72.68	6.07	1285.61
10	6.21	294.71	1.11	98.63	0.50	75.94	5.79	1283.74
Simulation tests	6.00	310.26	1.10	100.00	0.31	71.75	6.00	1334.14

The linear elastic deformation stage is of particular importance for the study of mechanical properties, including the elastic modulus. The *F*-*x* curves derived from the numerical simulation and physical tests conducted during this phase were extracted for fitting analysis. The discrepancy in the slope between the numerical simulation and physical tests is 5.21%, indicating a satisfactory fit. The fitted curves and results are presented in the blue box of **Figure 4B**, where *k_P_* represents the average slope of the physical tests and *k_S_* denotes the average slope of the numerical simulation tests. This indicates that the mechanical properties of the DEM model are similar to those of the actual CKS.

#### 3.3.2 Analysis of three-point bending physics experiments

**Figure 5:**
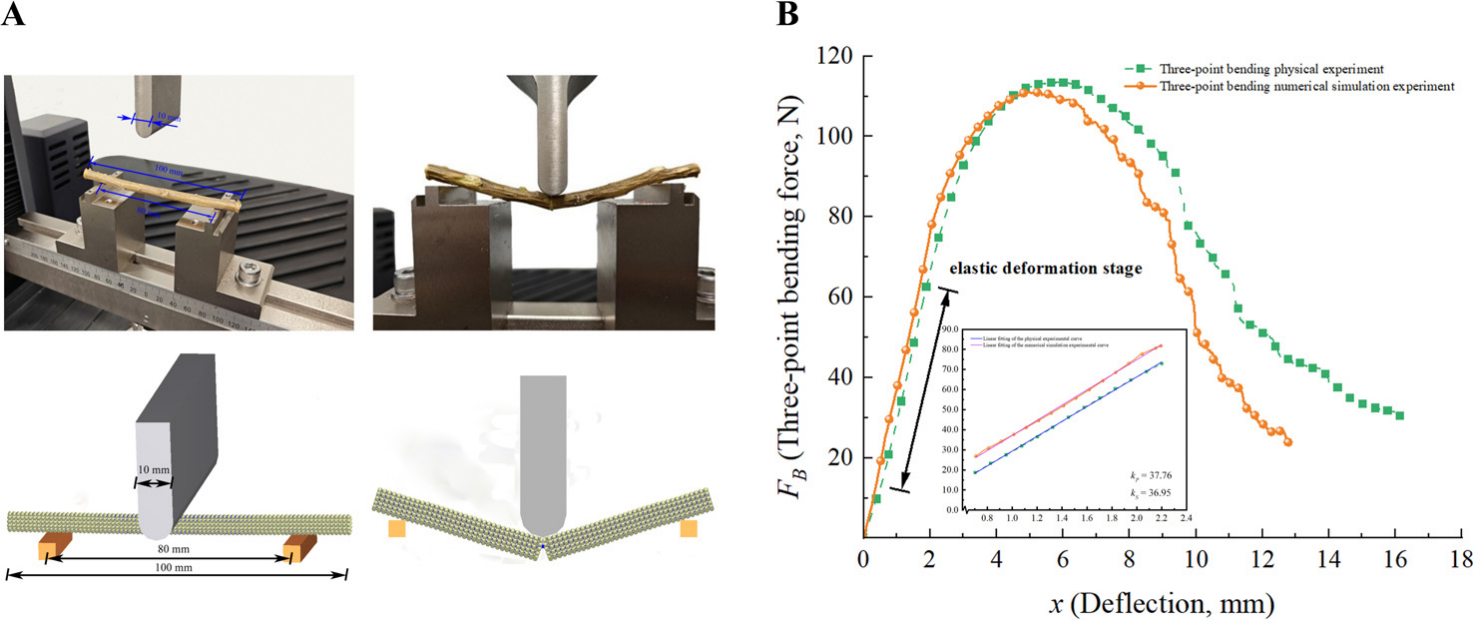
Analysis of three-point bending experiments. **(A)** The three-point bending physical and numerical simulation experiments of CKS; **(B)** The *F_B_*-*x* curves of physical and numerical simulation experiments.

**Figure 5A** shows the three-point bending physical and numerical simulation experiments of CKS. As illustrated in **Figure 5B**, the three-point bending test commences with an increase in bending force, resulting in the CKS entering the linear elastic deformation stage. During this phase, the CKS is capable of recuperating its deformation under the prevailing load. In an ideal scenario, the force and displacement should be proportional, in accordance with Hooke’s law. However, the force-displacement curve obtained from the experiment did not demonstrate a proportional relationship. This discrepancy may be attributed to the disparate physical properties of the phloem and xylem within the CKS, which renders it a heterogeneous material. However, the stem displayed characteristics consistent with the elastic stage. Subsequently, the CKS entered the yield stage, exhibiting substantial plastic deformation. The accumulation of various tissue cells resulted in a deceleration in the rate of increase in bending force. As the load continued to increase, reaching the maximum bending force, the stem entered the fracture stage. The bending force decreased markedly, resulting in the fracture of the CKS. At this moment, the bending force ceased to increase significantly with displacement, thereby marking the conclusion of the test. The three-point bending characteristics of the CKS are consistent with the test results of other stalks (Réquié et al., 2018). However, there are notable differences in the degree of force and the values of deflection deformation.

The two curves exhibit a general consistency in their respective trends. The numerical simulation yielded a value of *F_B_* of 111.83 N, exhibiting a 3.32% discrepancy with the target value of 115.67 N. This outcome substantiates the assertion that the DEM model of CKS effectively encapsulates the correlation between bending force and displacement. The overall trend of the numerical simulation curve is in leading of that of the physical test curve. This discrepancy can be attributed to the initial stage of the physical test, wherein the phloem of CKS, characterised by a high moisture content and soft texture, exerts a comparatively diminished influence on the rate of change in bending force (Guo et al., 2021). In contrast, the DEM model employed in the numerical simulation simplifies the material without distinguishing between the phloem and xylem, resulting in a more gradual change in bending force. In both the physical and numerical simulation tests, upon reaching the maximum bending force and entering the fracture stage, the change in bending force no longer follows a regular pattern of decrease. In the physical test, upon reaching the yield limit, the axial fibres of the CKS xylem enter the fracture stage. The accumulation of fibre cells results in a gradual reduction in bending force until the accumulated fibre cells reach their yield limit, at which point there is a sudden decline in bending force. This fracture stage then repeats the aforementioned process, resulting in a stepwise decrease in bending force. In contrast, the fracture stage of the DEM simulation curve demonstrates a more gradual decline in bending force in comparison to the physical test. This is due to the necessity of ensuring a reasonable computation time in the numerical simulation, which entails the simplification of the model and the reduction of the number of parallel bonds between particles to a level that is considerably lower than that observed in real fibre cells. Consequently, the accumulated particles exhibit diminished resistance to bending loads. During the linear elastic phase of the experimental procedure, the slope error of the *F*-*x* curve derived from the physical and numerical simulation tests is 2.15%.

#### 3.3.3 Analytical verification of axial tension and radial compression experiments

**Figure 6:**
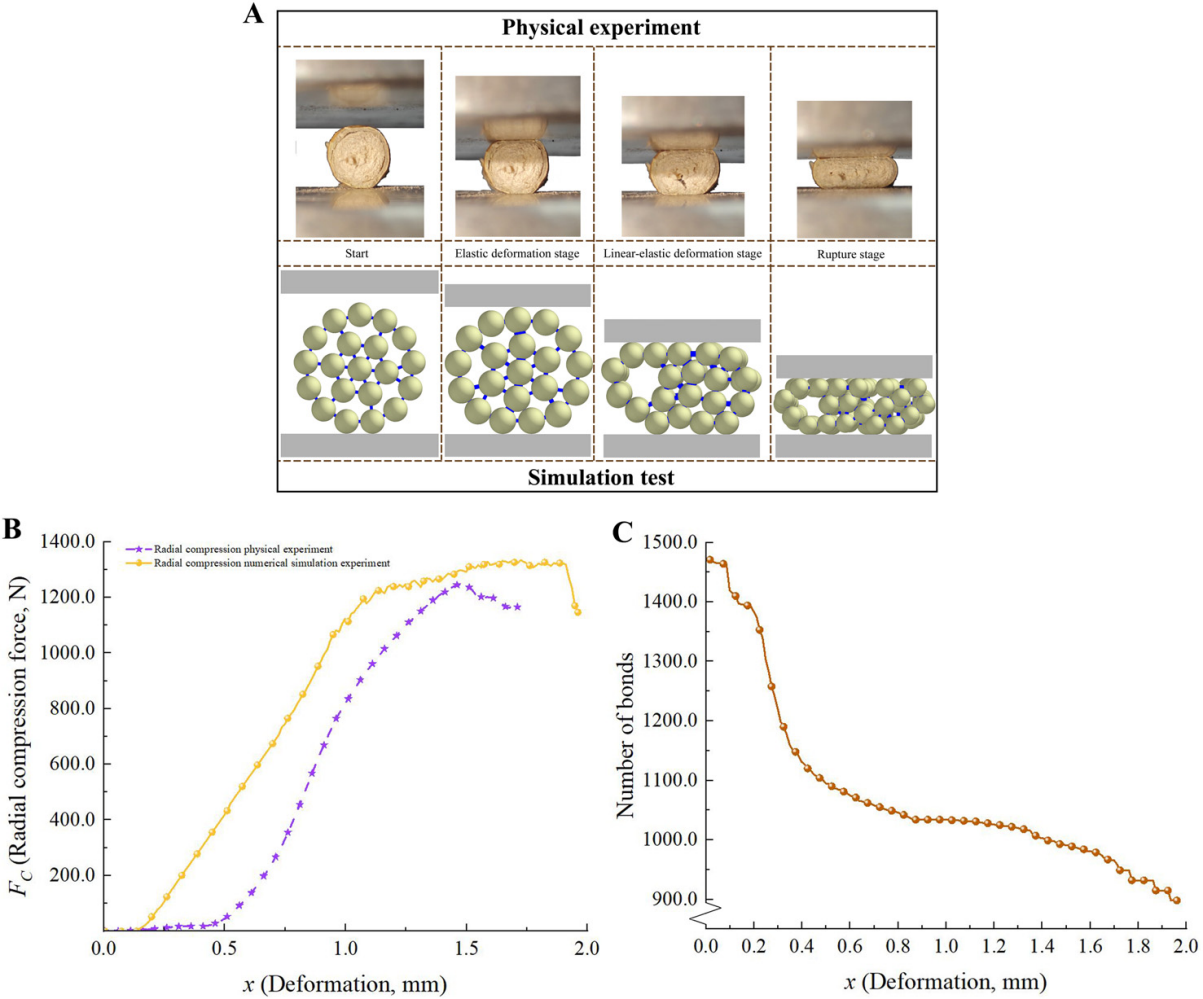
Analysis of radial compression experiments. **(A)** Performance of CKS in compression experiment; **(B)** The *F_C_*-*x* curves of physical and numerical simulation experiments; **(C)** Change process of total bond numbers.

**Figure 7:**
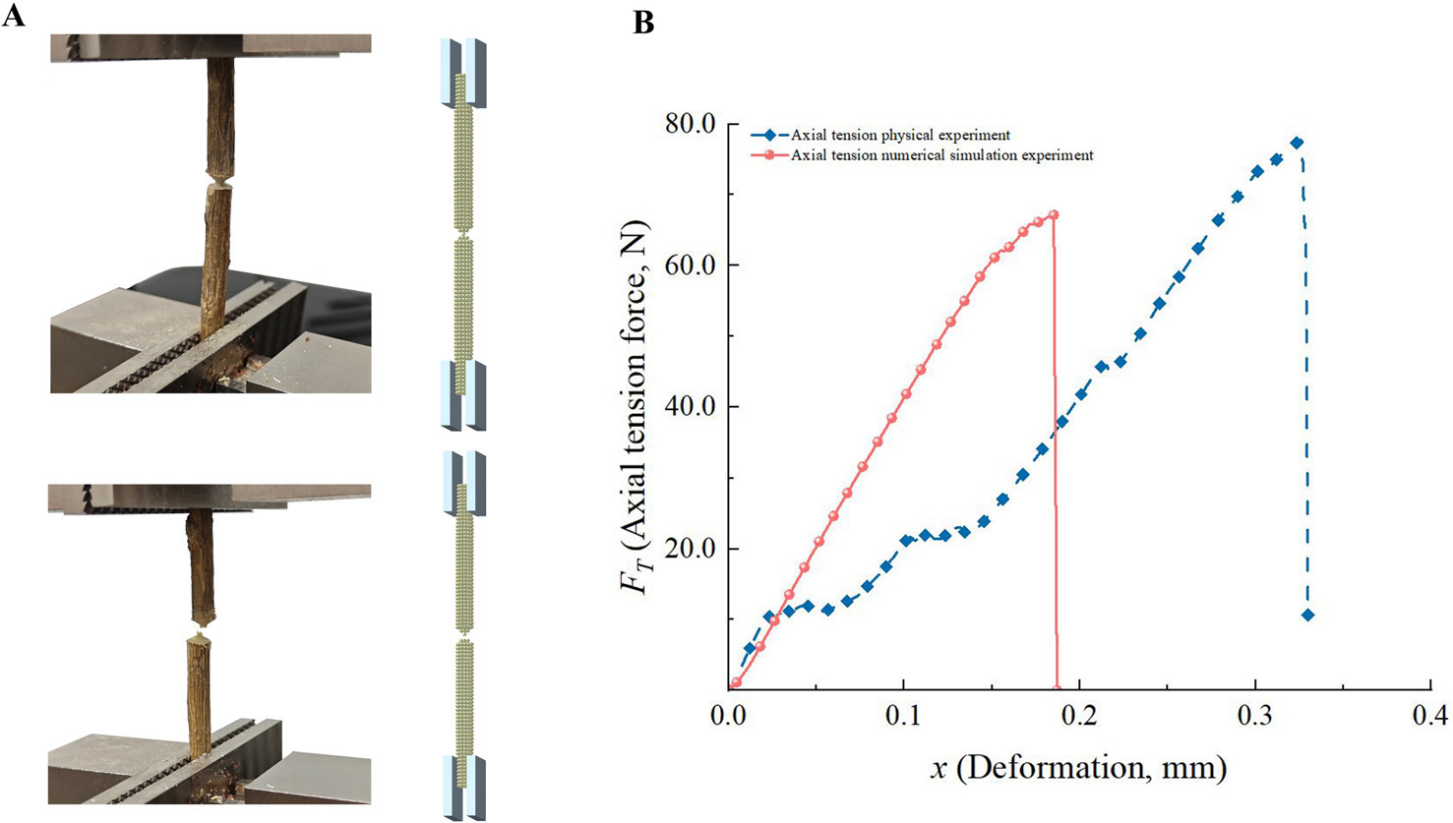
Analysis of axial tension experiments. **(A)** Axial tension experiments of CKS; **(B)** The *F_T_*-*x* curves of axial tension experiments.

**Figure 6** illustrates the processes of radial compression, while **Figure 7** depicts the processes of radial tension in the physical and numerical simulation tests. To guarantee the dependability of the axial tension experiments, the samples were subjected to a bespoke processing procedure. The ends of the samples were shaped into steps for easy clamping, and the middle part of the samples was made into a cylindrical shape (diameter was 1.10 ± 0.04 mm) with a smooth transition to avoid stress concentration (Li et al., 2024).

As illustrated in **Figure 6C**, it is evident that during the displacement range of 0-0.6 mm, the number of parallel bonds exhibits a pronounced decline. At this moment, the CKS is undergoing the expansion phase of cracking. Subsequent to 1.0 mm, the reduction in parallel bonds becomes more gradual, and the CKS gradually approaches the limit of its compressive strength. Upon reaching the maximum pressure, the CKS undergoes complete collapse, accompanied by the rupture of the parallel bonds within the model (Zhao et al., 2023).

The *F*-*x* curves for radial compression and axial tension in the physical tests exhibited trends that were generally consistent with those reported in previous studies, including those by (Guo et al., 2021; Zhao et al., 2023; Li et al., 2024). The *F*-*x* curves derived from the numerical simulation tests exhibited slight discrepancies compared to those obtained from the physical tests. This can be attributed to the use of uniform particles in the numerical model, which did not accurately represent the heterogeneous mechanical properties of the actual phloem and xylem. However, with regard to the maximum failure force, the discrepancy between the physical and numerical simulation tests was 4.37% for radial compression and 4.87% for axial tension, which is within an acceptable range.

#### 3.3.4 Cutting experiments analysis verification

**Figure 8:**
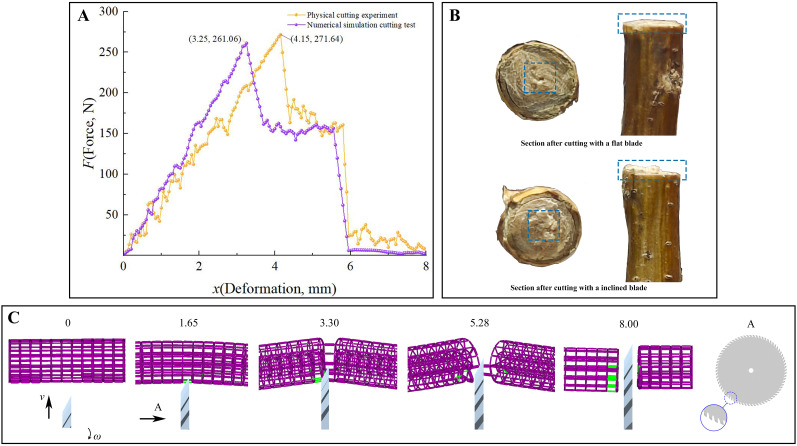
Analysis of cutting experiments. **(A)** The *F*-*x* curves of cutting experiments; **(B)** Sectional morphology of stems cut with different blades; **(C)** The progress of numerical simulation cutting test (purple bonds – the bond staus is normal, green bonds - the bond staus is breaking).

The *F*-*x* curves of the physical cutting process and the numerical simulation cutting process demonstrate a similar trend (**Figure 8A**). In the initial phase of the cutting process, the cutting force exhibited in the physical test displays greater fluctuations than in the numerical simulation. This is attributed to the more intricate internal structure of the stem, which differs from the discrete element model. As the tool advances further into the material, the cutting force reaches a maximum of 271.64 N, after which it declines rapidly. This occurs as the tool transitions from cutting the harder xylem to the softer phloem. However, the tool’s penetration also causes compression, which in turn gives rise to fluctuations in the cutting force during this stage. Subsequently, a second sharp decline in the cutting force is observed once the majority of the stem’s tissue has been cut, marking the complete severing of the stem and the conclusion of the test.

Furthermore, the numerical simulation cutting test also exhibits two sharp drops in cutting force. This phenomenon can be attributed to the exertion of a pushing action by the cutting tool on the parallel bonds during the cutting process. This action results in discontinuous fractures of the parallel bonds, preventing a smooth decrease in cutting force. An examination of the status of the parallel bonds reveals a greater number of broken bonds on the flat side in comparison to the inclined side, resulting in a more even and smooth cut on the flat side (**Figure 8C**). The results of the physical tests demonstrate that the actual CKS exhibits an uneven cross-section following cutting with a bevelled edge in comparison to a flat edge. This finding is in alignment with the performance observed in the numerical simulation tests (**Figure 8B**). The results indicate that the utilisation of a flat cutting method for stems is more advantageous for the subsequent growth of the stems and for the achievement of a smoother stubble after cutting.

The cutting performance was characterised by the shear stress *τ* (Equation 8). In this context, *F*_max_ represents the maximum cutting force, while *S_a_* denotes the cross-sectional area of the cut portion of the cutting specimen. The discrepancy between the numerical simulation results and the physical experiment was 3.89%, indicating that the discrete element model parameters had been calibrated reliably for subsequent studies.


(8) $$\tau =\frac{F_{\text{max}}}{S_{a}}$$

$$S_{a}=\pi r^{2}$$

### 3.4 Discussion

In this study, we found that the parallel bond parameter exerted a significant influence on the maximum bending force, while the contact parameter exerted a comparatively lesser effect on the *F*-*x* curve for three-point bending. Furthermore, the order of significance of the parallel bond parameters affecting stem failure force was found to be consistent with that reported in previous research (Zhao et al., 2023). Comparing and analysing physical tests with numerical simulation tests, the maximum failure force errors for the three-point bending, shear fracture, and radial compression tests were found to be small, and the *F*-*x* curve trends were observed to be consistent, same as previous studies (Sun et al., 2023; Zhao et al., 2023). However, the maximum failure force error for the axial tension test was smaller than that reported by (Guo et al., 2021), and the *F*-*x* curve trend was closer. The elastic deformation stage represented a crucial methodology for investigating the biomechanical properties of materials. Numerical simulation tests of three-point bending and shear fracture fitted equations at this stage with high accuracy and small errors between simulated and actual slopes. This provided a robust foundation for further studies on the elastic modulus of CKS. In the cutting test, both the inclined and flat sides of the tool and the parallel bond of the model were damaged. The inclined edge exhibited a tearing effect, resulting in more significant damage to the section. In contrast, the section after cutting by the flat edge displayed a relatively smooth surface (Li et al., 2024). This same performance was observed in the physical test. A comparison of the maximum cutting force and *F*-*x* between the physical and numerical simulation cutting tests reveals a small discrepancy. In comparison to other studies, this paper presented a comprehensive analysis of the biomechanical properties of CKS at both the micro and macro levels, and offered verification of the discrete element model from multiple perspectives. Furthermore, the *F-x* curves of all numerical simulation tests exhibited a leading trend compared to the physical tests. Additionally, the mechanical properties of xylem and phloem could not be adequately characterised by a single granular material. This limitation was to be addressed in future studies through the modelling of xylem and phloem separately. To date, there have been few studies on CKS. The use of DEM numerical simulation methods facilitated an understanding of the failure modes of the mechanical properties of real CKS at a microscopic level, thereby contributing to the subsequent in-depth study of tool-stem interaction mechanisms. It provided theoretical support for the study of CKS harvesting theory and harvesting machinery.

## 4 Conclusion

1. A DEM model of CKS based on the Hertz-Mindlin contact model was established, the parameters of which were calibrated. The optimal parameter combinations for each significant factor were then determined by Plackett-Burman design and response surface analysis experiments: shear stiffness per unit area is 3.56×10^9^ Pa, the bonded disk scale is 0.93 mm, the normal stiffness per unit area is 9.68×10^9^ Pa, the normal strength is 5.62×10^7^ Pa, the shear strength is 4.27×10^7^ Pa.
2. The numerical simulation tests for three-point bending, radial compression, axial tension, shear fracture, and cutting tests using the CKS model based on the aforementioned parameter combinations results with errors of 3.32%, 4.37%, 4.87%, 3.74%, and 3.89%, respectively, in comparison to their corresponding physical experiments. Additionally, the trend of *F*-*x* of the numerical simulation tests is approximately the same as the curve trend of the physical tests.
3. The cutting tests demonstrated that a smaller radial angle between the tool edge and the stem resulted in a more even and smooth cut surface. The DEM model of CKS established in this study effectively represents the biomechanical properties of real CKS, provides a basis for subsequent in-depth study of crop-machine interaction mechanisms, and guides the design and development of cutting devices for CKS harvesting machinery.

## Data Availability

The raw data supporting the conclusions of this article will be made available by the authors, without undue reservation.

## Glossary

*A*: The effective area of CKS, m^2^
*A_b_*: The cross-sectional area of the bonds, m^2^
*E*: Elastic modulus, Pa
*F*-*x*: Force and deformation curve
*F*_average_: The average value of *F_B_*, N
*F_C_*: Compression force, N
*F_B_*: Bending force, N
*F_T_*: Axial tensile force, N
*F_n_*, *F_n_’*: Normal adhesion forces of the bonds, N
*F_S_*: Shear force, N
*F_s_*, *F_s_’*: Tangential adhesion forces of the bonds, N
*G*: Shear modulus, Pa
*H*: CKS physical experiment drop height, m
*H’*: CKS DEM model simulation experiment drop height, m
*h*: CKS physical experiment post-crash rise height, m
*h’*: CKS DEM model numerical simulation experiment post-crash rise height, m
*I*: Moment of inertia of bonds, m^4^
*I_P_*: Polar moment of inertia of bonds, m^4^
*J*: Moment of inertia of CKS, m^4^
*k_n_*: Normal stiffness per unit area, N·m^−3^
*k_s_*: Shear stiffness per unit area, N·m^−3^
*L*: The effective length of CKS, mm
*L_b_*: The length of bonds, mm
Δ*L*: The change in the effective length, mm
*S*: Span of three-point bending experiment, mm
*S*_1_: CKS physics experiment incline movement distance, m
*S*_1_ *’*: CKS DEM model simulation experiment incline movement distance, m
*S*_2_: CKS physics experiment plane motion distance, m
*S*_2_ *’*: CKS DEM model simulation experiment plane motion distance, m
*V_n_*: Normal velocities of the particles, m·s^−1^
*V_t_*: Tangential velocities of the particles, m·s^−1^
*ω_n_*: Normal angular velocities of the particles, rad·s^−1^
*ω_t_*: tangential angular velocities of the particles, rad·s^−1^
*σ*_max_: Critical normal stress, Pa
*τ*_max_: Critical shear stress, Pa
*μ*: Poisson’s ratio
*ρ*: Density, kg·m^−3^
*θ*: The angle of the inclined plane of the static friction physical experiment, *°*
*θ’*: The angle of the inclined plane for static friction simulation experiment, *°*
*x*_1_: Coefficient of restitution between CKS and Steel
*x*_2_: Coefficient of static friction between CKS and Steel
*x*_3_: Coefficient of rolling friction between CKS and Steel
*δt*: Time step
*α*: The angle of the inclined plane of the dynamic friction physical experiment, *°*
*α’*: The angle of the inclined plane for dynamic friction simulation experiment, *°*
*V_n_*: Normal velocities of the particles, m·s^−1^
*V_t_*: Tangential velocities of the particles, m·s^−1^
CKS: Stem of *Caragana korshinskiil*
DEM: Discrete element method
