# Supplementary material for: Discrete element modelling and mechanical properties and cutting experiments of Caragana korshinskii Kom. stems
Source: Front Plant Sci. 2024 Oct 31;15:1457243. doi: 10.3389/fpls.2024.1457243 (PMC11560777; doi:10.3389/fpls.2024.1457243)
Supplement: Supplementary file 1 [file DataSheet1.docx]

**1. The supplementary material of section 2.1.2.2**

The particle radius was determined by physical and numerical simulation tests of AOR. Firstly, the AOR test of CKS was carried out using the cylinder lifting method, and the cylinder was raised at a speed of 50 mm/min to obtain the physical value of AOR (*β*). Then, standard spherical particles with particle radii of 3.0 mm, 1.0 mm, 0.5 mm and 0.25 mm were used to build the CKS model and numerical simulation tests of AOR were carried out at EDEM to obtain the simulated value of AOR (*β '*). The ratio of *β* and *β'* is the error value of simulation and physical test, and the simulation time is recorded to establish the mathematical models of particle radius and error, particle radius and simulation time as follows. It can be seen that the intersection point of the two straight lines is the particle radius of 0.55 mm, so the particle radius of the DEM model of CKS is selected as 0.55 mm.

| 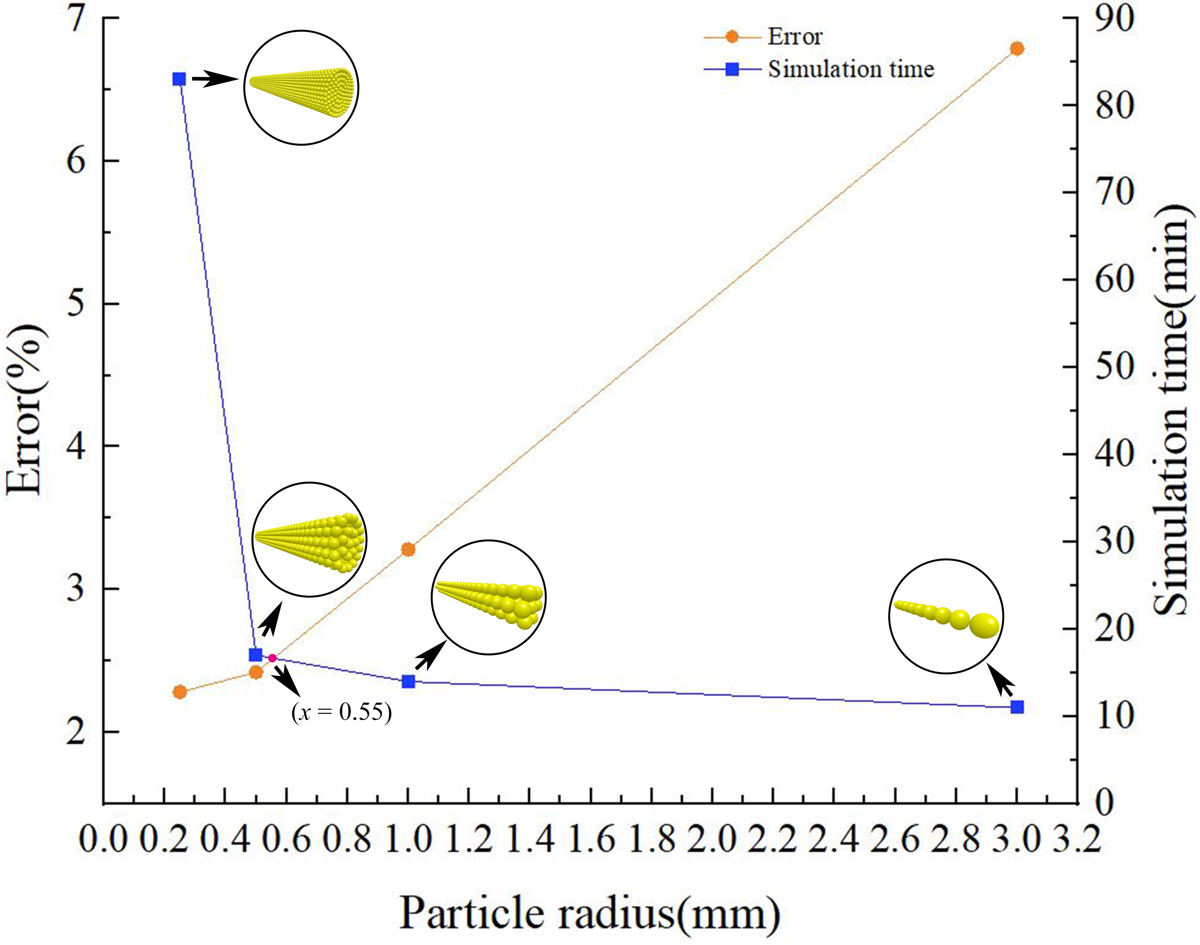 |
| --- |
| **Fig. S1.** Relation curves of AOR, simulation time and particle radius |

The contact radius should always be larger than the physical radius. If the bonding radius is too small, the bonding action will be very fragile, and if the contact radius is too large, the bonding between the particles cannot be created, usually, the contact radius should be 1.2 to 2 times of the physical radius (Zhang et al., 2019), and the final contact radius was determined to be 0.66 mm.

**2. The supplementary material of section 2.3**

The contact parameters of the materials include the coefficient of restitution, coefficient of static friction, and coefficient of rolling friction between materials and steel and between single materials. Their calibration process was shown in Fig. S1 and Fig. S2. Some of the characteristic parameters of the steel are used as the intrinsic parameters of the fixtures and brackets. These parameters can be extracted directly from the EDEM software library: *ρ* = 7820 kg⋅m^-3^, *μ* = 0.3, *G* = 8.19 × 10^10^ Pa.

| 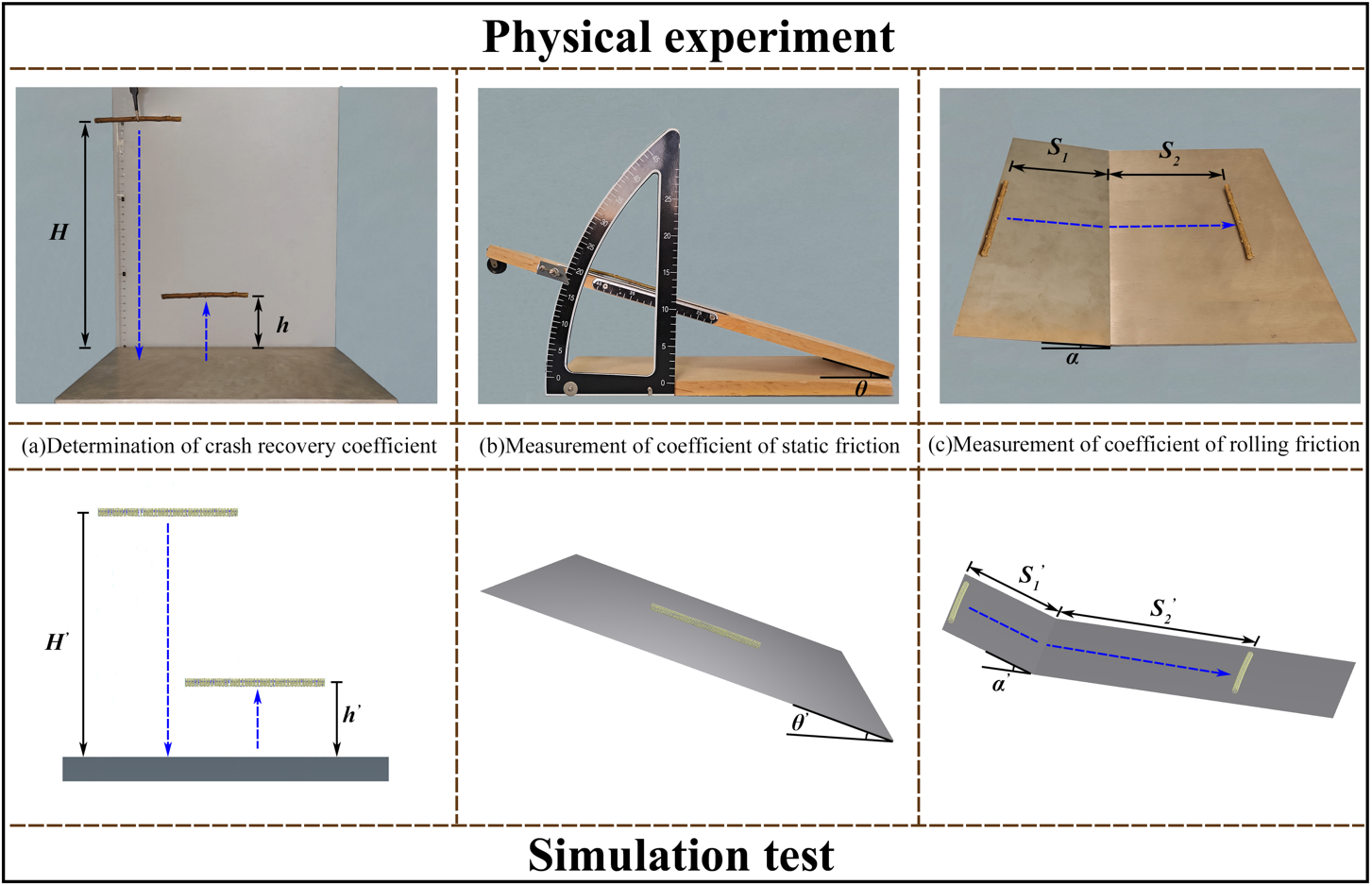 |
| --- |
| **Fig. S2.** Contact parameter measurement experiments. |

The CKS-steel contact parameters were first calibrated.

ⅰ) Coefficient of restitution

Since the cutting components of the CKS are generally made of steel, it is necessary to determine the coefficient of restitution between the CKS and steel by the free-fall method (Sun et al., 2023), which utilizes a high-speed camera to film the free-fall test process and record the data, as shown in Fig. S2(a), and the free-fall height of the CKS during the experiment was *H*. The rebound was achieved after the impact on the steel plate, and the maximum rebound height *h* was recorded by the high-speed video camera. The numerical simulation of this test was repeated in EDEM to keep *H'* and *H* consistent, and the coefficient of restitution was continuously adjusted until the maximum rebound height *h'* of the numerical simulation test was the same as that of the physical experiment, at which time the coefficient of restitution was the calibration value.

ⅰi) Coefficient of static friction

The coefficient of static friction between CKS and steel was determined using the tilt-slip experiment (Hu et al., 2023). The stem was placed flat on a stationary steel plate and the plate was slowly and uniformly lifted so that the material plate started sliding and the angle of inclination *θ* was recorded as shown in Fig. S2(b). The numerical simulation of this test was repeated in EDEM, and the static friction coefficient was adjusted so that the simulated value of the inclination angle *θ'* matched the physical value *θ*. The coefficient of static friction at this point is the calibrated value.

ⅰii) Coefficient of rolling friction

The coefficient of rolling friction between CKS and steel was determined using the inclined rolling experiment (Fan et al., 2022). That is, the CKS was released on an inclined plate with a preset angle *α*. The stem rolled downward along the inclined plane for a distance *S*_1_, and came to rest after rolling in the horizontal plane for a distance *S*_2_, as shown in Fig. S2(c), and a high-speed camera was utilized to record the experiment process. The test was simulated numerically in EDEM, keeping the inclined plane *α’* equal to *α*. The coefficient of rolling friction was adjusted so that the simulated values of the rolling distances *S*_1_*’* and *S*_2_*’* were consistent with the physical values of *S*_1_ and *S*_2_. The obtained coefficient of rolling friction is calibrated values.

Then the contact parameters between CKS-CKS were calibrated. The calibration was performed by AOR test (Zhang et al., 2023) with the following calibration steps: as in Fig. S3, the physical value of AOR of CKS (*β*) was first obtained by the cylinder lifting method. Then, the AOR test was reproduced in EDEM and with the contact parameter as the test factor, and the simulated value of (*β'*) of the AOR as the index, the Box-Behnken design test to establish the regression equation between contact parameters and AOR. The regression equation between contact parameters and AOR was established by Box-Behnken design test. Finally, the regression equation was solved using the AOR physical quantities as the response targets, and the optimal values of each parameter were obtained.

| 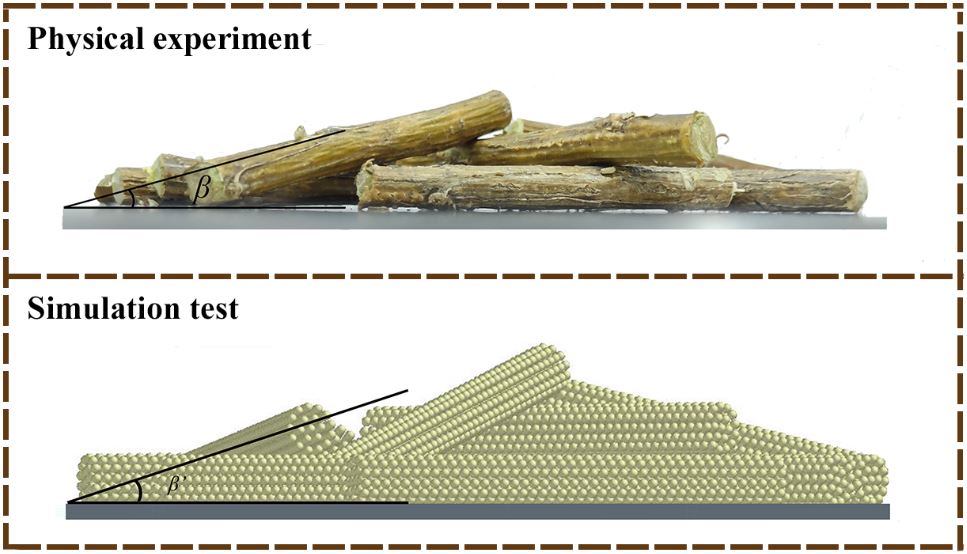 |
| --- |
| **Fig. S3.** AOR tests. |

The contact parameters were shown in Table S1.

| TABLE S1 The contact parameters of CKS. | | |
| --- | --- | --- |
| Parameters | | value |
| CKS-CKS | Coefficient of restitution | 0.40 |
|  | Coefficient of static friction | 0.23 |
|  | Coefficient of rolling friction | 0.34 |
| CKS-steel | Coefficient of restitution | 0.46 |
|  | Coefficient of static friction | 0.31 |
|  | Coefficient of rolling friction | 0.25 |

References

Fan, G. J., Wang, S. Y., Shi, W. J., Gong, Z. F., and Gao, M. (2022). Simulation parameter calibration and test of typical pear varieties based on discrete element method. *Agronomy.* 12, 1720. doi:10.3390/agronomy12071720.

Hu, Y., Xiang, W., Duan, Y.P., Yan, B., Ma, L., Liu, J. J., and Lyn, J. N.(2023). Calibration of Ramie Stalk Contact Parameters Based on the Discrete Element Method. *Agriculture-Basel.* 13 (5). 1070. doi:10.3390/agriculture13051070.

Sun, K., Yu, J. Q., Zhao, J. W., Liang, L. S., and Yu, Y. J. (2023). A DEM-based general modeling method and experimental verification for wheat plants in the mature period. *Comput. Electron. Agric.* 214, 108283. doi:10.1016/j.compag.2023.108283.

Zhang, F. W., Song, X. F., Zhang, X. K., Zhang, F. Y., Wei, W. C., and Dai, Fei. (2019). Simulation and experiment on mechanical characteristics of kneading and crushing process of corn straw. *Trans. Chin. Soc. Agric. Eng.* 35 (9), 58–65. doi:10.11975/j.issn.1002-6819.2019.09.007.

Zhang, S. W., Zhang, R. Y., Cao, Q. Q., Zhang, Y., Fu, J., Wen, X. Y., and Yuan, H. F. (2023). A calibration method for contact parameters of agricultural particle mixtures inspired by the Brazil nut effect (BNE): The case of tiger nut tuber-stem-soil mixture. *Comput. Electron. Agric.* 212, 108112. doi:10.1016/j.compag.2023.108112.
